# Expression Optimization of Anti-CD22 scFv-Apoptin Fusion Protein Using Experimental Design Methodology

**DOI:** 10.22034/ibj.22.1.66

**Published:** 2018-01

**Authors:** Solmaz Agha Amiri, Najmeh Zarei, Somayeh Enayati, Mohammad Azizi, Vahid Khalaj, Soraya Shahhosseini

**Affiliations:** 1 Department of Pharmaceutical Biotechnology, School of Pharmacy, Shahid Beheshti University of Medical Sciences, Tehran, Iran; 2 Department of Medical Biotechnolgy, Biotechnology Research Center, Pasteur Institute of Iran, Tehran, Iran; 3 Department of Pharmaceutical Chemistry, School of Pharmacy, Shahid Beheshti University of Medical Sciences, Tehran, Iran

**Keywords:** Recombinant protein, Single-chain antibodies, Fusion proteins, *E. coli*

## Abstract

**Background:**

Design of experiments is a rapid and cost-effective approach for optimization of recombinant protein production process. In our previous study, we generated a potent dual-acting fusion protein, anti-CD22 scFv-apoptin, to target B-cell malignant cell lines. In the present investigation, we report the effect of different variables on the expression levels of this fusion protein.

**Methods:**

Four variables (cell optical density at induction, IPTG concentration, induction temperature, and induction time) were tested using experimental design.

**Results:**

Our findings demonstrated that among the examined variables, only the induction time had a significant positive effect on the protein expression yield.

**Conclusion:**

Experimental design was successfully applied in this study. The optimized condition obtained in the current study can be applied in future commercial production of this novel fusion protein.

## INTRODUCTION

Recombinant pharmaceutical proteins such as antibody fragments offer many advantages over traditional drugs. There are several platforms for production of target recombinant proteins, including bacterial, yeasts, and mammalian expression systems[1,2]. Although the selection of the expression host is dependent on the nature of the target protein, the *E. coli* expression system is the preferred host for screening and even initial development of commercial recombinant products[3,4]. The advantages of using *E. coli* as a bacterial expression system include the rapid growth rate to high cell density, inexpensive production substrate, and easy genetic manipulation and transformation[4,5]. The efficiency of the recombinant protein expression in this host is related to the genetic characteristics of the *E. coli* strain, expression vectors and the target heterologous protein. In addition, manipulation of the expression process to optimize protein production is essential to enhance the yield of the protein[6]. The classical method for optimization of a protein expression is to change one factor at a time while keeping other factors constant. This method is often ineffective because changing one factor at a time does not represent the combined effect of the involved variables. In addition, it is a time-consuming process and needs a large number of experiments[6,7]. To overcome this problem, the best approach is to use the statistical design of experiments, which allows simultaneous evaluation of many more variables at lower cost with a few experimental trials[6-8]. In view of this consideration, the goal of this work was to improve the expression of a previously described fusion protein, anti-CD22 scFv-apoptin, in *E. coli*[9], using experimental design.

## MATERIALS AND METHODS

### Strains and plasmids

The recombinant anti-CD22 scFv-apoptin protein was successfully expressed in *E. coli* BL21 (DE3) with IPTG induction using the expression vector, pET-28a (+). The cloning and expression procedures have been described in detail in our previous study[9].

### Design of experiment

The effects of four variables in two levels, lower (-1) and higher (+1), which have been related to induction conditions (cell concentration at induction, IPTG concentration, induction temperature, and time) were investigated using a general factorial design (10^2^). All experimental designs and data analysis were investigated using Design-Expert 7.0 software. The significance of each variable effect and the interaction of variables on the induction were determined using analysis of variance (ANOVA), and the *P* value lower than 0.05 was considered as statistically significant. A positive effect at significant level implies that alteration from lower (-1) to the higher value (+1) resulted in the improvement of the target response, and also a negative effect means a decrease in the target response from (-1) to (+1) levels of a variable.

### Bacterial growth and protein expression conditions

The growth condition for the expression of the recombinant scFv-apoptin has been described before [9]. Briefly, *E. coli* BL21 (DE3) harboring the expression construct was grown in 5 ml Luria-Bertani medium containing the appropriate antibiotic. At the

OD of induction (0.5 or 1.5 according to the experimental design), the inducer (IPTG) was added at the concentration of 0.1 or 1 mM depending on the experimental design. Subsequently, at the induction phase, the culture was incubated at 25 or 37 °C for 4 or 24 h based on the experimental design. All the 16 different experimental conditions were presented in Table 1.

**Table 1:** Factorial design and response (production of scFv-apoptin)

Condition	Induction absorbance (ab_ind_)	IPTG (mM)	Expression temperature (°C)	Induction time (h)	scFv-apoptin (mg/ml)
1	0.5 (-1)	0.1 (-1)	25 (-1)	4 (-1)	0.320
2	0.5 (-1)	0.1 (-1)	37 (+1)	4 (-1)	0.110
3	0.5 (-1)	1.0 (+1)	25 (-1)	4 (-1)	0.220
4	0.5 (-1)	1.0 (+1)	37 (+1)	4 (-1)	0.053
5	0.5 (-1)	0.1 (-1)	25 (-1)	24 (+1)	0.260
6	0.5 (-1)	0.1 (-1)	37 (+1)	24 (+1)	0.240
7	0.5 (-1)	1.0 (+1)	25 (-1)	24 (+1)	0.210
8	0.5 (-1)	1.0 (+1)	37 (+1)	24 (+1)	0.410
9	1.5 (+1)	0.1 (-1)	25 (-1)	4 (-1)	0.120
10	1.5 (+1)	0.1 (-1)	37 (+1)	4 (-1)	0.340
11	1.5 (+1)	1.0 (+1)	25 (-1)	4 (-1)	0.120
12	1.5 (+1)	1.0 (+1)	37 (+1)	4 (-1)	0.180
13	1.5 (+1)	0.1 (-1)	25 (-1)	24 (+1)	0.460
14	1.5 (+1)	0.1 (-1)	37 (+1)	24 (+1)	0.360
15	1.5 (+1)	1.0 (+1)	25 (-1)	24 (+1)	0.390
16	1.5 (+1)	1.0 (+1)	37 (+1)	24 (+1)	0.320

+1 describes the highest level of variable, and -1 represents the lowest level of variable.

### SDS-PAGE and protein expression analysis

The induced cultures (2 ml) were centrifuged at 10,000 ×g for 10 min at 4 °C and the pellets were resuspeded in an appropriate buffer, as described previously[9] and disrupted by sonication. Following centrifugation and resuspension, total protein concentration of cell extract was measured by Bradford method, and 20 µg of each cell extract was loaded on a 12% SDS-PAGE gel. The protein bands were stained with Coomassie brilliant blue R-250, and the densitometry was used to analyze the recombinant scFv-apoptin corresponding band using Quantity One 4.62 software (Bio-Rad laboratories, Hercules, CA, USA).

## RESULTS AND DISCUSSION

Experimental design is a multivariate technique that enables to estimate the effect of changing more than one variable at a time on a distinct response. In addition, this statistical technique permits to characterize the statistically significant variables and interaction between them[6,7,10]. As the majority of heterologous proteins are expressed intracellularly in *E. coli*, growing the expression host to the high cell-density leads to increased yield of the final product. Fermentation condition is one of the most important factors that affects the bacterial growth, and it could be optimized in a way to provide high cell densities during the production process. Design of experiment is a methodical approach used for rapid optimization of different protein expression processes[7, 11].

Anti-CD22 scFv-apoptin is a dual anti-cancer fusion protein that have been produced as a possible drug candidate against B-cell malignancies. This protein has been expressed in *E. coli* mainly in insoluble form[9]. In the present study, the effects of different variables on the scFv-apoptin production were investigated using a panel of designed experiments.

As indicated in Table 1, the difference of the protein expression levels in 16 independent experiments demonstrated that the examined variables have significant effects on the production of scFv-apoptin. Statistical analysis revealed that only induction time had a significant influence on the results of the experimental design with a positive effect (Table 2). This means that the maximum response was obtained in the higher induction time. The other three variables, absorbance at induction, induction temperature, and IPTG concentration showed no significant effect (Table 2).

**Table 2:** ANOVA for effects on the scFv-apoptin expression

Factors	scFv-apoptin expression	*p* value
ab_ind_	+0.029	0.2682
Time (h)	+0.074	0.0129*
IPTG (mM)	-0.019	0.4595
Temperature (°C)	-5.438E-003	0.8320

A negative signal indicates a negative effect of the variable on the response, while a positive signal shows a positive effect

* (*p* < 0.05)

In the case of temperature, a negative effect was seen (Table 2), showing a minimum expression level at higher temperature. Correlation between temperature and post-induction time has been reported by other researchers[6,8]. They found that longer incubation times at higher temperatures have unfavorable effects on the protein production, especially in soluble form. Although scFv-apoptin was expressed in the form of inclusion bodies, in this experimental design, the same interaction between higher incubation time and lower temperature was seen (data not shown). Another variable with negative effect was IPTG concentration (Table 2). This phenomenon could be explained as a result of toxic characteristics of IPTG[7,12].

Cell absorbance at induction showed a positive effect on this experimental design (Table 2). Induction could be done at variable cell concentrations in early or late logarithmic phase, depending on the protein and expression systems. The high level of recombinant protein expression may inhibit host cell growth, due to its toxicity and restriction of the metabolic sources. To overcome this problem, one strategy is growing cells to high cell density prior to addition of the inducer[6,7].

**Fig. 1:**
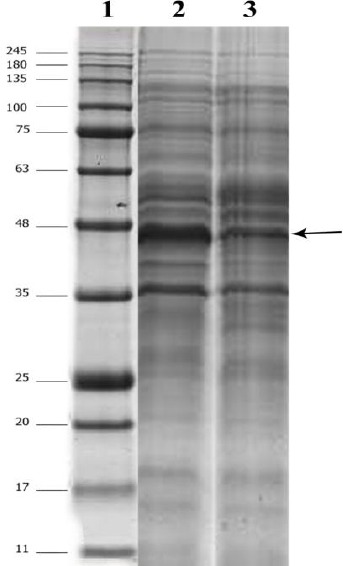
SDS-PAGE analysis of the recombinant scFv-apoptin. Total protein (20 µg) was loaded in each lane in each sample of cell lysate. Lane 1, protein size marker (kDa); lane 2, scFv-apoptin expressed at abs_ind_ = 1.5, IPTG = 0.1 mM, temperature = 25 °C, and time = 24 h; lane 3, scFv expressed under the same condition except the time of induction that was 4 h. The recombinant protein (44 kDa) is shown by arrow.

Thus, as presented in Table 1 and Fig. 1, the expression was greater at OD of induction 1.5, time of 24 h, IPTG concentration of 0.1 mM and temperature 25 °C.

The analysis presented in this work showed that experimental design is a valuable tool for investigating the effect of the different factors on the production of the scFv-apoptin protein and also the improvement of the production levels of this protein for using in downstream application.
